# The role of autophagy and ferroptosis in sensorineural hearing loss

**DOI:** 10.3389/fnins.2022.1068611

**Published:** 2022-12-12

**Authors:** Ying Sun, Shengyu Zou, Zuhong He, Xiong Chen

**Affiliations:** Department of Otorhinolaryngology-Head and Neck Surgery, Zhongnan Hospital of Wuhan University, Wuhan, China

**Keywords:** autophagy, ferroptosis, ferritinophagy, sensorineural hearing loss, hair cell

## Abstract

Hearing loss has become a common sensory defect in humans. Because of the limited regenerative ability of mammalian cochlear hair cells (HCs), HC damage (caused by ototoxic drugs, aging, and noise) is the main risk factor of hearing loss. However, how HCs can be protected from these risk factors remains to be investigated. Autophagy is a process by which damaged cytoplasmic components are sequestered into lysosomes for degradation. Ferroptosis is a novel form of non-apoptotic regulated cell death involving intracellular iron overloading and iron-dependent lipid peroxide accumulation. Recent studies have confirmed that autophagy is associated with ferroptosis, and their crosstalk may be the potential therapeutic target for hearing loss. In this review, we provide an overview of the mechanisms of ferroptosis and autophagy as well as their relationship with HC damage, which may provide insights for a new future in the protection of HCs.

## Introduction

As a common sensory disorder in humans, hearing loss affects 1.5 billion people around the world (WHO, 2021). Sensorineural hearing loss (SNHL), an important type of hearing loss, refers to pathological changes in the cochlear hair cells (HCs), auditory nerves, or central auditory system. Ototoxic drugs, aging, and noise are common pathogenic factors of hearing loss that can cause damage or loss of HCs (Mills and Going, 1982). Unfortunately, adult mammalian cochlear HCs are not capable of regeneration; consequently, irreversible damage to HCs can result in permanent deafness (Zine et al., 2021). Protecting HCs is, therefore, a key focus of auditory research. However, the detailed mechanism underlying cochlear HC damage remains largely unknown.

In recent years, autophagy and ferroptosis have attracted much attention, and the modulation of autophagy and ferroptosis is a very attractive potential approach for the future treatment of SNHL. Autophagy is a highly conserved degradation system in eukaryotic cells that can remove damaged organelles and macromolecular proteins and be induced by reactive oxygen species (ROS) accumulation (Boya et al., 2018). In the auditory system, especially in HCs, previous studies reported the relationship between autophagy and hearing loss (Guo et al., 2021; Zhao et al., 2021), specifically, that moderately activating autophagy can reduce HC damage in both cochlear HCs and HEI-OC1 cells (a mouse auditory cell line) after neomycin or gentamicin injury (He et al., 2017). Ferroptosis is a non-apoptotic mechanism of cell death that mainly relies on the iron-mediated production of ROS and follows plasma membrane damage (Dixon and Stockwell, 2014). Generally, ferroptosis accompanies lipid peroxidase, impaired antioxidant capacity, and increased intracellular iron accumulation (Tang et al., 2021). New research has found that the development and progression of many diseases are related to ferroptosis (Lin et al., 2022; Wang K. et al., 2022). Recently, several studies have revealed the link between ferroptosis and hearing loss and proved that the inhibition of ferroptosis may alleviate HC damage (Hu et al., 2020; Zheng et al., 2020; Jian et al., 2021).

In this paper, we review recent studies about the role of autophagy and ferroptosis in HC damage. We also focus on the molecular regulatory mechanism of autophagy and ferroptosis *in vitro* and *in vivo*. Additionally, in this review, we discuss the regulatory impact of autophagy on ferroptosis and further discuss whether regulating ferroptosis through autophagy could be a potential measure for the protection of HCs.

## The role of autophagy in hair cell damage

Autophagy is not only a physiological process of degradation for long-lived proteins and damaged organelles in eukaryotic cells but also an important adaptive mechanism in the response to cellular stress (e.g., infection, poisoning, and hypoxia) (Chen et al., 2017). Under normal physiological conditions, autophagy is essential for the maintenance of cell homeostasis, while under some stress conditions, autophagy will be activated and promoted. Autophagy is divided into macroautophagy, microautophagy, and chaperone-mediated autophagy (Morel et al., 2017). Generally speaking, macroautophagy, as the main process for cells to remove damaged organelles and other related fragments, is referred to as “autophagy” (Feng et al., 2014). Autophagy takes place throughout the dynamic development of cells and plays the function of a “double-edged sword” (Wei et al., 2012). In other words, while autophagy confers a pro-survival effect, allowing cells to fight against cellular stressors such as excess ROS (Chen et al., 2017), under certain circumstances, excessive autophagy can also cause autophagic cell death (Clarke and Puyal, 2012).

### The role of autophagy in age-related hearing loss

Age-related hearing loss (ARHL) is the most common form of aging in the auditory system and clinically manifests as progressive bilateral symmetrical hearing loss (Bowl and Dawson, 2019). As a major cause of sensory disturbances, ARHL limits social interaction among older individuals and thus leads to cognitive function decline, depression, anxiety, and loneliness (Jafari et al., 2021). Oxidative stress theory is the most common hypothesis regarding aging (Martin et al., 1996). With increasing age, the production of ROS increases, while body antioxidant capacity gradually decreases (Fu et al., 2018). The level of autophagy decreases with age (Youn et al., 2020), and moderate upregulation of autophagy can promote the survival of aging HCs by scavenging damaged mitochondria, which may suggest that autophagy impairment is a key factor in ARHL (Dong et al., 2021; Gumeni et al., 2021). Moderately promoting autophagic activity may, therefore, advance the viability of aging HCs.

Recently, some molecules have been reported to regulate the level of autophagy in ARHL. MicroRNAs (miRNAs) are a kind of short, endogenous, and highly evolutionarily conserved single-stranded RNAs. miRNAs can regulate the expression of target genes by inducing the degradation and inhibiting the translation of target mRNA. miR-34a blocks autophagy flux by inhibiting the expression of the autophagy-related gene 9A (ATG9A) and promotes the death of senescent HEI-OC1 cells (Pang et al., 2017). Additionally, miR-34a mediates autophagy through more than one target. In HEI-OC1 cells, siRNA was used to downregulate the expression of Sirtuin 1 (SIRT1), which can impair autophagy by significantly decreasing the conversion of microtubule- associated protein 1 light chain 3 (LC3) I to LC3 II and influencing the deacetylation of ATG9A, inducing the accumulation of p62 and HEI-OC1 cell death (Pang et al., 2019). One study suggested that miR-34a targeted SIRT1, protected cochlear HCs, and delayed ARHL by regulating mitochondria-specific autophagy (mitophagy) (Xiong et al., 2019). In addition, Forkhead box G1(FOXG1) protects HCs from oxidative damage by activating autophagy during cell senescence (He et al., 2021).

As a selective kind of autophagy, mitophagy plays an important role in ARHL pathological changes. Mitophagy refers to the process of selectively degrading damaged mitochondria and maintaining mitochondrial homeostasis. Since mitochondria are the “energy factories” and free radical metabolic centers in cells, dysfunction caused by mitochondrial damage leads to metabolic disorders of ROS. Excessive ROS can cause oxidative damage to mitochondrial lipids, DNA, and proteins, making mitochondria produce more ROS and eventually triggering apoptosis (Ashrafi and Schwarz, 2013). However, to prevent cellular damage, mitophagy will be activated to preserve a population of healthy mitochondria. Researchers found that mitophagy is involved in aging HCs. Prohibitin 2 (PHB2), a protein receptor on the intima of mitochondria, is exposed when the mitochondrial outer membrane is ruptured and then interacts with the autophagy protein LC3 II to activate mitophagy. Yu et al. (2021) found that PHB2 expression was increased along with mitophagy activation in mice with ARHL, indicating that PHB2 may be involved in mitophagy. However, the exact mechanism underlying the role of PHB2 in ARHL is unclear and thus needs to be explored further (Yu et al., 2021). BCL2 interacting protein 3 like/Nip3-like protein X (BNIP3L/NIX) is a protein located in the outer mitochondrial membrane that participates in mitophagy by promoting the formation of autophagosomes to engulf target mitochondria (Li et al., 2021). BNIP3L/NIX was significantly downregulated in both *in vivo* and *in vitro* models of aging, while overexpression of BNIP3L/NIX alleviated HC senescence by promoting mitophagy (Kim et al., 2021).

### The role of autophagy in drug-induced hearing loss

Chemotherapeutic agents (e.g., cisplatin) and aminoglycosides (e.g., neomycin and gentamicin) are two major classes of ototoxic drugs. These drugs can cause bilateral and irreversible SNHL. Because of their side effects, the application of ototoxic drugs is limited, and hearing loss prevention following ototoxic drug treatment has long attracted researchers’ attention. ROS have been identified as one of the classical causes of SNHL mediated by cisplatin and aminoglycosides (Gentilin et al., 2019; Fang et al., 2022) and can be generated after ototoxic drug treatment (Liu et al., 2016; Li et al., 2018). If ROS are generated in excess, they will damage the antioxidant defense capacity of HCs and induce cell apoptosis (Gentilin et al., 2019).

Autophagy is a common cellular response when cells are exposed to stress stimuli such as ototoxic drugs. Previous studies showed that ROS can induce autophagy in auditory cells after cisplatin and aminoglycoside injury (Hirose et al., 1997; Liu et al., 2021). Autophagic activity increases significantly in HCs following treatment with neomycin or cisplatin (Levano and Bodmer, 2015; He et al., 2017). The activation of autophagy has been observed to promote HC survival after neomycin treatment, and blocking the activation of autophagy can increase ROS levels and induce HC apoptosis (He et al., 2017). Therefore, exploring the effect of autophagy on HC protection along with the underlying mechanism may have therapeutic implications for the prevention and treatment of ototoxic drug-induced hearing loss.

Autophagy is regulated by many factors during ototoxic drug damage. The overexpression of YTHDF1 (the YT521-B homology N6-methyladenosine RNA binding protein) could enhance the activation of autophagy by promoting the translation of the autophagy-related gene ATG14 (autophagy-related gene 14), while a deficiency thereof suppressed autophagy and increased the loss of HCs after cisplatin damage (Huang et al., 2022). Overexpression of TFEB (transcription factor EB), a transcription factor, activated autophagy in cochlear HCs and HEI-OC1 cells and subsequently alleviated cisplatin-induced apoptosis (Li et al., 2022). Upregulation of autophagy by phosphorylation of the Ser9 site of GSK-3β(glycogen synthase kinase-3β), a multifunctional serine/threonine protein kinase, could attenuate cisplatin-induced ototoxicity, which may be related to the activation of the WNT/β-catenin signaling pathway (Liu et al., 2019). PINK1 (phosphatase and tensin homolog induced putative kinase 1)/Parkin-mediated mitophagy can inhibit mitochondrial proteotoxicity and maintain mitochondrial protein homeostasis. Research reported that in HEI-OC1 cells, neomycin attenuated PINK1/Parkin-mediated mitophagy by promoting ATF3 (activating transcription factor 3) expression, and damaged cochlear HCs could be partially rescued when mitophagy was induced (Zhang et al., 2022). After cisplatin treatment, ROS aggravates the damage and promotes the activation of PINK1/Parkin-related mitophagy to clear dysfunctional mitochondria and inhibit the activation of JNK (c-Jun N-terminal kinase)-related apoptotic pathways to protect HEI-OC1 cells against cisplatin-induced damage (Yang et al., 2018).

### The role of autophagy in noise-induced hearing loss

Noise is one of the main causes of SNHL. Following noise exposure, the accumulation of ROS contributing to the pathogenesis of noise-induced loss of sensory HCs is well accepted. Noise-induced stress increases the level of ROS in outer HCs and then leads to HC damage, which is the key to metabolic injury (Hill et al., 2016). In addition, ROS induce inflammation by producing cytokines and damage cochlear HCs (Wakabayashi et al., 2010). However, ROS can also induce autophagy to perform cellular defense functions (Vernon and Tang, 2013).

Following noise exposure, autophagy is triggered to limit pathological changes to HCs. Miao et al. (2021) found that two autophagy metabolites, phosphatidylethanolamine (PE) and phosphatidylcholine, were significantly decreased in the plasma samples of patients suffering from noise-induced hearing loss (NIHL). They confirmed that PI3K (phosphatidylinositide 3-kinase), AKT (protein kinase B), and ATG5 (autophagy-related gene 5) were significantly downregulated in NIHL patients by using real-time quantitative PCR (Miao et al., 2021). Yuan et al. (2015) found that increasing autophagy activity by using rapamycin can inhibit ROS accumulation and reduce noise-induced HC loss (Yuan et al., 2015). In contrast, treatment with either the autophagy inhibitor 3-methyladenine or LC3 II siRNA can exacerbate noise-induced HC loss and NIHL (Fang et al., 2022). Recently, pejvakin has been reported to regulate autophagy in NIHL. Pejvakin is a peroxisome-associated protein from the gasdermin family that helps to maintain the normal three-dimensional ciliated ladder structure and mechanical transduction of HCs (Kazmierczak et al., 2017). In response to sound overstimulation, pejvakin promotes pexophagy (the autophagic degradation of peroxisomes) by recruiting LC3 II directly and protects the auditory HCs against oxidative stress (Defourny et al., 2019). Based on the above studies, the importance of autophagy in NIHL is worthy of further exploration.

## The role of ferroptosis in hair cell damage

Dixon et al. (2012) identified a novel form of regulated cell death, denoted “ferroptosis.” The morphology and biochemistry of ferroptosis differ from other forms of cell death. Morphologically, mitochondrial cristae loss and outer membrane rupture are characteristic signatures of ferroptotic cells (Xie et al., 2016). Biochemically, ferroptosis is characterized by its association with the accumulation of iron and the subsequent production of ROS, as well as lipid peroxidation (Xie et al., 2016). Once the ability of the antioxidant system to scavenge overexpressed ROS is exceeded, oxidative stress will occur, resulting in lipid peroxidation, which damages cellular structures and leads to cell death. The labile iron pool, moreover, can facilitate a Fenton reaction and peroxidize polyunsaturated fatty acids (PUFAs) to generate lipid peroxides, resulting in plasma membrane rupture and eventually, cell death (Stockwell, 2022). In brief, iron-dependent ROS production results in lipid peroxidation and ultimately leads to membrane damage, which is the core mechanism of ferroptosis. ROS produced by the Fenton reaction, PUFAs, and lipid peroxidation can promote ferroptosis, but the system Xc^–^/glutathione(GSH)/glutathione peroxidase 4(GPX4) axis can inhibit ferroptosis by influencing the redox status of cells (Liang et al., 2022).

Since ferroptosis was discovered, some evidence has shown the close relationship between ferroptosis and many pathologies. For example, ferroptosis is widely believed to function as a tumor-suppression mechanism. Phosphoglycerol dehydrogenase, by binding and regulating PCBP2 [Poly(rC) binding protein 2] protein expression to promote the mRNA stability of SLC7A11 (the catalytic subunit of system Xc^–^), can inhibit cell ferroptosis and ultimately promote the malignant progression of bladder cancer (Shen et al., 2022). The ferroptosis inducer dihydroartemisinin intensively strengthens the cytotoxicity of cisplatin for pancreatic ductal adenocarcinoma cells, which may act as a promising therapeutic strategy for drug-resistant tumors (Du et al., 2021).

Similarly, ferroptosis also plays an important role in HC injury. Neomycin induced intracellular Fe^2+^ increases and lipid peroxidation in the HEI-OC1 cell line and cochlear HCs (Zheng et al., 2020). After cisplatin treatment, the mitochondria in HEI-OC1 cells showed mitochondrial shrinkage and loss of mitochondrial crests—typical characteristics of ferroptotic cells (Hu et al., 2020). Moreover, ferroptosis may be a cause of auditory cortex degeneration, leading to central presbycusis. Levels of acyl-CoA synthetase long-chain family member 4 (ACSL4) and transferrin receptor 1 were increased in the auditory cortex of rat models that mimic aging induced by D-galactose, and removing excess iron from cells by deferoxamine treatment inhibited ferroptosis and delayed cellular senescence (Chen et al., 2020). However, the role of ferroptosis in aging HCs has not been reported.

As mentioned above, ferroptosis occurs when lipid ROS production exceeds the antioxidant capability. Glutathione peroxidase 4 (GPX4) can convert lipid hydroperoxides to lipid alcohols by using reduced GSH as a cofactor, reducing ROS-induced damage to cells (Loscalzo, 2008). GPX4 is regarded as the robust central regulator and lethal signal of ferroptotic cell death, and the inactivation or absence of GPX4 can cause the accumulation of lipid peroxides. Inhibiting GPX4 will lead to a redox homeostasis imbalance, eventually resulting in ferroptotic cell death. Neomycin significantly decreased the expression of GPX4 in cochlear HCs (Zheng et al., 2020). Hu et al. (2020) found that the expression of GPX4 was inhibited by cisplatin, and ROS accumulated in HEI-OC1 cells. These findings may suggest that the balance of redox homeostasis in ototoxic drug-damaged HCs is disrupted, which could promote ferroptotic cell death. Nuclear factor erythroid 2-related factor 2 (Nrf2) is a key transcription factor in the antioxidant response. When the organism is under oxidative stress, Nrf2 dissociates from Keap1 (Kelch-like ECH-associated protein 1) and binds to the antioxidant response elements of target genes such as GPX4, activating the transcription of antioxidant genes. Positively regulating the Nrf2-GPX4 axis can prevent ferroptosis in carbon tetrachloride-induced acute liver injury in mice (Zhao et al., 2022). Knockdown of Nrf2 facilitated erastin-induced cell destruction in HepG2 cells, implying a pivotal role of Nrf2 against ferroptosis (Dai et al., 2021). However, knockout of Nrf2 substantially inhibited cisplatin-induced HEI-OC1 cell death by decreasing transferrin receptor 1 protein levels and increasing GPX4 protein levels (Wang W. et al., 2022). These results suggest that the role of Nrf2 in ferroptosis regulation may be tissue-specific.

Excessive lipid peroxidation can induce ferroptosis in cells. How lipid peroxidation is generated during ferroptosis depends on the enzyme system that promotes phospholipid peroxidation. For example, ACSL4 catalyzes PUFAs in the membrane structure to produce corresponding coenzyme A, which then forms lipid peroxides through a series of downstream reactions and eventually leads to cell disintegration (Doll et al., 2017). ACSL4 was a key enzyme in arachidonic acid-induced ferroptosis. He et al. (2022) found that cisplatin-treated HCs showed significant enrichment in the arachidonic acid metabolic pathway by metabolomics assays. Researchers found that inhibiting the expression of ACSL4 could reduce the content of lipid peroxides and protect HCs (He et al., 2022). These findings indicate that HCs treated with cisplatin experience lipid metabolism disorders, which, if prevented by intervention, can resist ferroptosis and reduce the destructive effect of cisplatin on HCs. Thus, ACSL4 may be a key factor contributing to cisplatin sensitivity in HCs and a potential therapeutic target for SNHL.

Several ferroptosis inhibitors may allow for potent and selective therapeutic measures for preventing HC damage. Liproxstatin-1 and ferrostatin-1, which are aromatic amine antioxidants, inhibit lipid peroxidation directly by free radical capture (Zilka et al., 2017). A study demonstrated that liproxstatin-1 protected HCs from aminoglycoside-induced ototoxicity by inhibiting ferroptosis in HEI-OC1 cells and neonatal mouse cochlear HCs (Zheng et al., 2020). Similar effects were also observed for ferrostatin-1 in damaged HCs (Hu et al., 2020). Regarding their mitigating effect on ototoxicity, ferroptosis inhibitors may be expected to be used to protect HCs from ototoxic drugs. In addition, from the perspective of HCs damage, ferroptosis has only been reported in ototoxic drug-induced hearing loss, and whether this pathway is involved in ARHL and NIHL needs to be further explored.

## The relationship between autophagy and ferroptosis

The relationship between autophagy and ferroptosis has attracted much attention. As universally acknowledged, autophagy is often involved in ferroptosis. Increasing evidence indicates that ferroptosis requires autophagy machinery for its execution. For example, erastin, a ferroptosis inducer, can activate autophagy, while knockout of some autophagy-related genes significantly inhibited ferroptosis induced by erastin (Hou et al., 2016). Autophagy can lead to the accumulation of iron ions and lipid peroxidation, eventually promoting ferroptosis.

Ferritinophagy refers to a selective autophagy process that manifests as the movement of ferritin to lysosomes and its degradation, releasing free iron. Through quantitative proteomics pair analysis of all the proteins in autophagosomes, Mancias et al. (2015) discovered a specific protein, the nuclear receptor coactivator 4 (NCOA4), that is closely related to ferritin and so were the first to discover and name the process of ferritinophagy. Highly enriched in autolysosomes, NCOA4 mediates the targeted recognition of ferritin by autophagosomes by binding to ferritin (Gao et al., 2016). Ferritinophagy plays an important regulatory role in organism pathology. When ferritinophagy is over-induced, Fe^2+^ overload in the cytoplasmic matrix promotes lipid peroxidation, resulting in cell membrane damage and ferroptosis (Ajoolabady et al., 2021). However, blocking autophagy or knocking out NCOA4 can inhibit the accumulation of lipid ROS, so preventing the eventual occurrence of ferroptosis (Gao et al., 2016).

**FIGURE 1:**
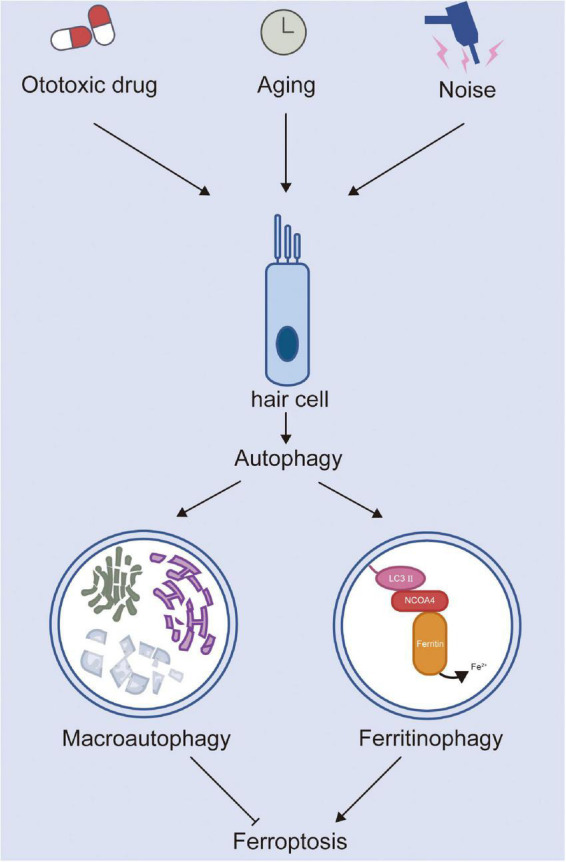
The roles of autophagy and ferroptosis in hair cell injury.

Therefore, studying the mechanism of NCOA4-mediated ferritinophagy and its pathophysiological role in different diseases may provide avenues for treatment. Ito et al. (2021) established a mouse model of heart failure by using the method of transverse aortic constriction. Their results showed that, compared with the control group, the accumulation of free iron and lipid peroxidation were inhibited in the hearts of mice with knockout of NCOA4. Further, the degree of left ventricular dilatation was reduced and cardiac function was improved, indicating that activating ferritinophagy can lead to the development of heart failure. Compound 9a was used to block the interaction between NCOA4 and ferritin heavy chain 1 to inhibit ferritinophagy and block ferroptosis, thus significantly improving the brain damage of rats with ischemic stroke (Fang et al., 2021). Similarly, cisplatin treatment promoted ROS-induced lipid peroxidation and caused iron accumulation by activating NCOA4-medicated ferritinophagy (Jian et al., 2021). In summary, most studies have shown that the upregulation of ferritinophagy can promote the occurrence of ferroptosis, while the downregulation of ferritinophagy can inhibit ferroptosis (Figure 1). Currently, ferritinophagy is less studied in the field of auditory diseases, and targeting ferritinophagy may be a promising therapeutic strategy for the treatment of SNHL. In addition, selective autophagy processes such as lipophagy (Bai et al., 2019), clockophagy (Yang et al., 2019), and chaperone-mediated autophagy (Wu et al., 2019) also cause lipid peroxidation and subsequent ferroptotic cell death and may be potential targets of SNHL.

The relationship between autophagy and ferroptosis, however, is controversial. Subjecting cells to oxidative stress and injury can activate their self-protection mechanism. Autophagy may protect cells against ferroptotic cell death. Sorafenib could induce both apoptosis and ferroptosis in Desmoid-type fibromatosis cells. Furthermore, using the autophagy inhibitor hydroxychloroquine could enhance sorafenib-induced cytotoxicity. These results show that autophagy may have a pro-survival function through the inhibition of ferroptosis and apoptosis (Schut et al., 2022). Similarly, Zhao et al. (2020) reported that 15-lipoxygenase binds to PE to produce an oxidation product, 15-hydroperoxyeicosatetraenoic acid, which can lead to pro-ferroptotic cell damage and activate the pro-survival autophagy pathway to limit cell damage. Therefore, it seems to be a closely regulated mechanism between autophagy and ferroptosis. More research is needed to reveal the molecular mechanism between autophagy and ferroptosis and provide new potential therapeutic targets for hearing loss.

## Conclusion

Generally, autophagy plays a dual role in HC damage. Moderately increased autophagy can help maintain intracellular homeostasis by reducing oxidative stress, while autophagic cell death can occur under other conditions. Although many molecules that can regulate autophagy have been reported, research has been limited to external and animal experiments, and few clinical trials have examined the impact of autophagy on HCs.

Ferroptosis is widely investigated in both physiologic and pathogenic processes but has not been extensively studied in the auditory field. At present, it is reported that ototoxic drugs (e.g., neomycin and cisplatin) can induce ferroptosis in HCs, but it remains unclear whether ferroptosis engages in aging and noise-induced HC damage. Regulating ferroptosis may, therefore, be a promising strategy in SNHL therapy. However, additional studies on ferroptosis and its involvement in SNHL are needed to identify the corresponding therapeutic targets.

Increasingly, the relationship between autophagy and ferroptosis has appealed to researchers. An increasing number of studies have revealed that ferritinophagy-mediated ferroptosis is involved in the occurrence and development of neurodegenerative disease, reperfusion injury, and cancers (Santana-Codina and Mancias, 2018). While ferritinophagy is a worthy target for the treatment of SNHL, its complete molecular mechanism and pathophysiological process in SNHL still call for further study. Clarification of the crosstalk between autophagy and ferroptosis would not only favor a comprehensive understanding of cell death pathways but also provide us with new ideas for the future treatment of SNHL.

## Author contributions

XC and ZH conceived and designed the study and reviewed and edited the manuscript. YS and SZ wrote the manuscript. All authors have read and approved the submitted version of the manuscript.
